# SPR Sensing: From Biomolecular Interactions to Cell-Based Analysis

**DOI:** 10.3390/bios16060332

**Published:** 2026-06-10

**Authors:** Petia Genova-Kalou, Evdokiya O. Hikova, Todor Kereziev, Petar T. Kolev, Vihar Mankov, Hristo Kisov, Anna Atanasova, Georgi L. Dyankov

**Affiliations:** 1 National Center of Infectious and Parasitic Diseases, 44A Gen. Stoletov Blvd., 1233 Sofia, Bulgaria; petia.d.genova@abv.bg; 2 Institute of Optical Materials and Technologies “Acad. J. Malinowski” (IOMT), Bulgarian Academy of Sciences (BAS), 109 Acad. G. Bonchev Str., 1113 Sofia, Bulgaria; p.kolev@iomt.bas.bg (P.T.K.); vmankov@iomt.bas.bg (V.M.); alalova@iomt.bas.bg (A.A.); gdyankov@iomt.bas.bg (G.L.D.); 3 Central Laboratory of Applied Physics, Bulgarian Academy of Sciences, 61 Sankt Petersburg Blvd., 4000 Plovdiv, Bulgaria; todorkrz@gmail.com (T.K.); hristokisov@iomt.bas.bg (H.K.)

**Keywords:** surface plasmon resonance, cell-based analysis, proliferation, morphology, cytotoxicity, pharmacokinetics

## Abstract

Surface plasmon resonance (SPR) is a key tool for quantifying biomolecular interactions, and its use in studying interacting components outside cellular systems is well-established. Over the past 20–25 years, cell-based SPR techniques have emerged, with the promise of precise detection of molecular interactions within their normal physiological environment. Research on a wide variety of biological samples, which requires the detection of numerous parameters, has led to the development of a broad range of SPR techniques. This review aims to trace the chronological development of these techniques and the factors that have driven them. In this context, particular focus is given to grating-coupled SPR applied to cell assays. Its specific capabilities are examined, and the respective advantages and disadvantages of other SPR techniques are discussed based on the results obtained from studying specific biological objects. Finally, we venture to predict the promising SPR techniques, as well as the areas of application in which significant results can be expected.

## 1. Introduction

Cell-based assays are a valuable tool for providing a functional assessment of molecules in their physiological environment. Furthermore, they provide information on the integrated behaviour of living cells in response to external stimuli. Consequently, they have become a key technology in modern biology for drug discovery and biosensing. Their applications include drug screening, signalling pathway studies, assessment of morphological changes or proliferation, and evaluation of cytotoxicity and selectivity.

Once it was demonstrated [1,2,3] that the efficiency of biochemical reactions observed in cell-based assays could be assessed using SPR analysis, the technique became widely applied. SPR-based cell-based assays were first applied for detecting changes in whole cells upon exposure to external stimuli. Later, they were adapted for detecting specific cellular responses to interactions with molecules or drugs [4,5,6]. The main advantage of using SPR in cell-based assays is the ability to observe cell interactions in real time without the need for labels.

Among label-free optical methods, SPR is one of the most powerful techniques for analysing living cells. SPR occurs when p-polarised light interacts with the electrons of a thin metal layer, typically gold or silver, at a specific angle [7]. When the angle of incidence and wavelength are optimal, the energy of the photons is transferred to the free electrons in the metal, producing collective oscillations. These oscillations generate a surface wave (plasmon wave) that is extremely sensitive to variations in the refractive index at the metal–dielectric interface. When applied in cell-based assays, where cells are seeded onto a surface, the SPR technique is capable of detecting minute variations in the refractive index resulting from changes in mass, alterations in cell morphology, and biochemical signalling processes in living cells. As a result of cell adhesion, growth, or structural modifications, the interaction of these cells with the evanescent electromagnetic field of the surface plasmon causes a variation in the optical characteristics of the reflected light. This modification of the resonance condition, which can be experimentally detected as a change in angle, wavelength, intensity or phase, produces the SPR signal.

The first article to review the application of SPR in cell-based assays was [8]. It summarised the available research at that time, focusing on the use of SPR in cell-based assays for diagnosing allergies and cancer. It also provided information on some SPR techniques. There are currently many reviews, but they tend to focus on a particular SPR technique or object of study.

**Figure 1:**
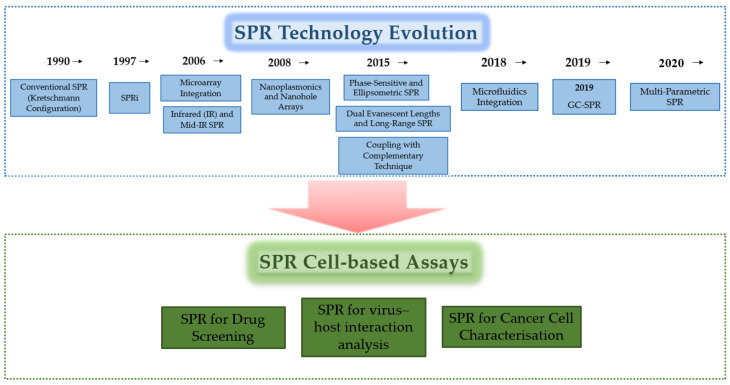
The chronological development of the various SPR techniques (upper rectangle) used in cellular research across different fields (bottom rectangle).

In this review, we trace the chronological development of various SPR platforms, analyse their performance and offer our insights into future developments. Figure 1 shows the approximate years in which the first reports on the application of each SPR technique for cell analysis appeared. We have attempted to identify the first article and the most recent review on each SPR technique, though we do not claim to be exhaustive given the vast database that was reviewed manually. We evaluate the advantages and disadvantages of each technique and comment on the research subjects. As Figure 1 shows, grating-coupled SPR has recently been applied to cell analysis. In the context of the chronological development of SPR cell assay techniques and their capabilities, we assess the importance, effectiveness and potential of grating-coupled GC-SPR techniques. Given the unique properties of the GC-SPR technique and the results obtained thus far, we anticipate a growing interest in its application to cell analysis. This could lead to technological breakthroughs in terms of spatial resolution and selectivity, particularly accelerating the development of new drugs.

## 2. SPR Techniques Applied for Cell-Based Assay

Surface plasmon resonance (SPR) detects total changes in the refractive index caused by molecular binding and/or mass redistribution near the cell membrane, enabling the observation of living systems [9]. Advances in SPR technologies, from the Kretschmann method to imaging and multiparametric detection and the most recent SPR techniques, have expanded the scope of research in molecular biology, pharmacology and biomedical engineering. This section traces the chronological development of SPR configurations as applied to cell-based assays, outlines their respective advantages and limitations, and concludes with a comparative assessment of recent advances in the context of their future applications.

### 2.1. Conventional SPR (Kretschmann Configuration, 1990–2025)

The classical SPR (cSPR) setup, introduced by Otto and Kretschmann in the late 1960s, uses a thin noble-metal film (typically gold) deposited on a prism through which p-polarised light excites surface plasmons under total internal reflection. In the 1980s, the method was adapted for biomolecular interaction analysis, becoming a cornerstone of label-free biosensing [7].

**Figure 2:**
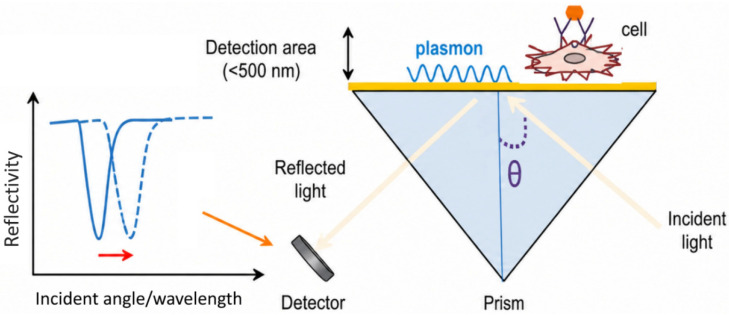
Kretschmann configuration and principle of measurement—cells are cultured on the surface of the SPR sensor. The arrow indicates the direction of the incident and reflected light beams. The SPR signal is modulated by cell adhesion, growth, or structural modifications. Adapted from Ref. [9].

During the 1990s, the method was adapted for cell-based assays with the addition of temperature stabilisation and noise suppression. Figure 2 shows the principle of measurement. Since then, significant effort has been devoted to promoting stable cell adhesion and minimising non-specific effects [2,8].

The conventional prism-based configuration remains the most widely adopted form of SPR for biosensing application:

- Advantages: it offers robust, quantitative kinetic measurements, straightforward optical alignment, and well-established calibration routines; it has moderate penetration depth (150–400 nm) that matches the typical adhesion distance between the cell membrane and the substrate, making it ideal for monitoring adhesion, detachment, and ligand-induced morphological shifts [2].
- Disadvantages: it is unsuitable for studying heterogeneous or patterned cell layers; it is sensitive to temperature fluctuations and refractive index drift.

Despite these limitations, the Kretschmann design remains the most widely used for quantitative cell studies without labelling thanks to its improved stabilisation optics and biocompatible coatings [10].

### 2.2. SPR Imaging (SPRi, 1990s–2000s)

**Figure 3:**
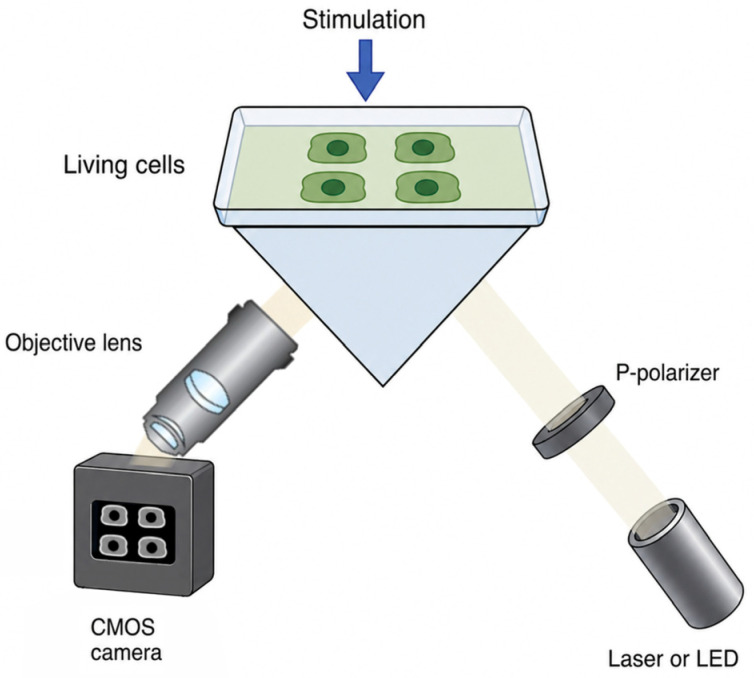
Structure of the SPR imaging instrument. The monochromatic light is collimated; observation is performed at a fixed angle of incidence; obtained images and light intensity of indicated areas are analysed by suitable software. Adapted from Ref. [18].

The capabilities of conventional SPR were expanded to include image analysis of the entire field across the sensor surface by replacing the photodiode detector with a charge-coupled device (CCD) or complementary metal-oxide-semiconductor (CMOS) camera. The first SPR imaging observation was carried out by examining the morphology of monolayers under a microscope [11]. The principle of SPRi is illustrated in Figure 3. Thus, reflectivity in cell cultures seeded at various locations on the sensor could be analysed, as well as adhesion heterogeneity and cell spreading dynamics.

SPRi was initially employed in the study of DNA-DNA interactions [12]. Since then, this method has been used to visualise drug responses, wound healing, and collective cell migration in real time. Using artificial intelligence-based methods to process images obtained from high dynamic range detectors has significantly improved sensitivity and reproducibility by correcting optical artefacts. A thorough and detailed review of the capabilities of SPRi and its areas of application can be found in [13,14]. SPRi two-dimensional field of view allows for the simultaneous visualisation of cell populations and multiple microarray spots on the same patterned sensing chip [15]. One advantage of SPRi is the ability to analyse drug-induced morphological changes in hundreds of cells. The sensitivity of the analysis depends heavily on the number and size of the pixels. Although software enhancement can increase sensitivity, a trade-off must be made between spatial and temporal resolution. The development of SPRi technology is summarised in [16,17], along with an analysis of its advantages and disadvantages.

### 2.3. Microfluidics Integration (2000s–2010s)

The need for rapid, automated detection using small sample volumes necessitates the integration of surface plasmon resonance (SPR) sensor technology with microfluidic methods [19]. This integration improves the precision and reproducibility of the assay [20], enabling dynamic dosing and gradient generation, both of which are essential for chemotaxis and cytotoxicity studies. Microfluidic SPR chips have been employed to investigate cell adhesion, receptor activation and drug-receptor kinetics at the level of individual cells [21]. Reported sensitivity is 7.7 × 10^−6^ RIU and time resolution is 2.5 s.

A detailed review of the various types of integrated SPRi/microfluidic systems, their respective advantages and disadvantages, and potential applications is provided in [22]. Continuous improvements in microchannel design and surface passivation have enhanced cell viability and enabled long-term monitoring under flow conditions. However, maintaining laminar flow, ensuring uniform cell exposure and preventing shear-induced stress remain technical challenges. Flow integration can be challenging in certain SPR formats (e.g., grating-coupled), sometimes necessitating workarounds instead of true continuous flow, which complicates time-resolved analyses and sample processing.

### 2.4. Microarray Integration (2000s)

Microchip biosensors have led to a technological breakthrough in the rapid, multiplex detection of surface bioaffinity interactions. Subsequently, microchips have been successfully applied in the fields of genomics, genetic testing, gene expression and drug discovery. The integration of SPRi technology with microchips has proven to be a successful alternative to conventional fluorescence-based microchips. This approach was first applied to study the morphology of phospholipid monolayers [23]. The SPR-specific measurements of changes in the refractive index were subsequently utilised to assess bioaffinity adsorption onto biopolymer microarrays [24]. In angular or spectral detection mode, SPRi allows for the simultaneous detection of multiple points. This requires multiplexed flow control to be implemented, a process that is optimised through the use of microfluidics [25].

### 2.5. Nanoplasmonics and Nanohole Arrays (Early 2000s)

**Figure 4:**
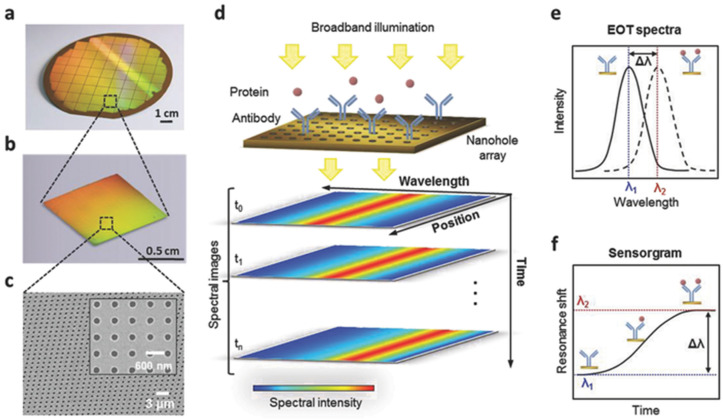
Nanoplasmonics and nanohole arrays: (**a**) plasmonic nanohole arrays fabricated uniformly on a thin gold film; (**b**) sensor chip for analysis; (**c**) SEM image: nanoholes with diameter 200 nm, distance centre to centre: 600 nm; (**d**) chip functionalised with biorecognition elements. The yellow arrows indicate the direction of the broadband illumination incident on the nanohole array, while the black arrows indicate the spectral response. The colors represent the spectral intensity distribution at different time points during the binding process.; (**e**) spectral wavelength shift in the EOT resonance peak; (**f**) sensogram obtained in real time. Reproduced with permission from [28]. Copyright © 2018 John Wiley & Sons.

The capabilities of SPR technology have been significantly expanded through the use of exceptional optical transmission (EOT) [26]. Figure 4 shows the principle providing localised field enhancement by nanoplasmonic sensors, comprising nanoholes, nanoprisms, and nanodiscs. This expansion enhances local electromagnetic fields and sensitivity. The objective is achieved through the implementation of nanoplasmonic substrates and nanohole arrays. Nanoscale field confinement has been demonstrated to enhance the signal-to-noise ratio, thereby facilitating the detection of minimal cell membrane deformation, endocytosis, and cytoskeletal activity [27].

However, this increased precision comes at a significant cost, as it requires the use of electron beam lithography, a process that is known to be costly. Additionally, the nanostructured surface has been demonstrated to influence cell adhesion, thereby introducing variability in biological responses [27]. That said, the diminutive dimensions of nanoplasmonic structures facilitate their integration into lab-on-a-chip and single-cell systems, thereby conferring unparalleled potential for single-cell and subcellular biosensing [28].

### 2.6. Phase-Sensitive and Ellipsometric SPR (Mid-2000s)

Plasmon resonance is accompanied by a sharp change in the phase of reflected light. Phase-sensitive SPR measures this change. According to some reports, this measurement method leads to 100 times greater detection sensitivity [29]. It is particularly valuable for detecting low-mass analytes and ionic fluctuations during cell signalling. Phase-sensitive SPR biosensor based on Mach–Zehnder configuration has been used for efficient targeted drug screening and an excellent sensitivity of 10^−6^ RIU has been reported [30].

In order to monitor kinetics of cell adhesion and cellular response to an external stimulus, a grating-coupled SPR sensor utilising phase interrogation has been employed [31]. The sensitivity achieved was 300/RIU.

However, phase-based methods are optically complex and susceptible to thermal and mechanical noise. Consequently, they are primarily employed in specialised laboratory settings rather than for routine cell analysis.

Ellipsometric SPR measurement methods represent a reasonable compromise between practicality and accuracy. When applied to live stem cells, this method provides reliable information on the response to methadone treatment [32].

### 2.7. Infrared (IR) and Mid-IR SPR

IR excitation has been shown to broaden the evanescent field of the plasmonic wave from approximately 400–520 nm in the 600–800 nm range to 1–2 μm in the 2–2.5 μm range [33,34]. This enhances sensitivity to processes occurring near and beneath the plasma membrane in whole cells, while simultaneously reducing photodamage and phototoxicity. This facilitates real-time work with living cells.

**Figure 5:**
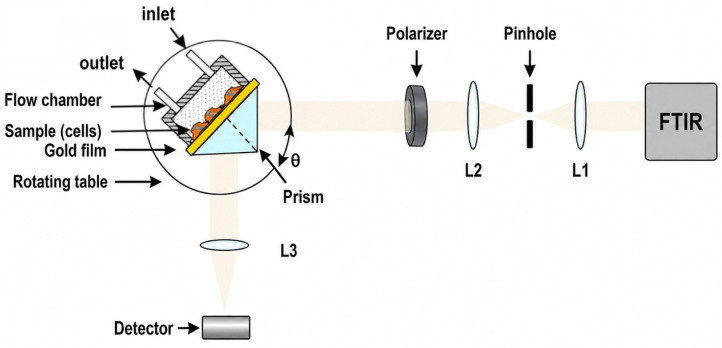
FTIR-SPR experimental setup; cells are cultured onto the sensing surface and are illuminated with a collimated polarised IR light beam emitted from the FTIR spectrometer. Adapted from Ref. [33].

Figure 5 shows the experimental setup. Living cells were cultured onto a senser surface and were illuminated with an IR light beam emitted from the Fourier Transform Infrared Spectroscopy (FTIR) spectrometer. Integrating SPR with FTIR spectrometry enabled detection in the 0.8–1.2 μm range in multi-wavelength mode. The biological applications include quantitative determination of D-glucose in solution without labelling, monitoring of glucose uptake by erythrocytes, monitoring of cholesterol incorporation into HeLa membranes, and tracking of transferrin-induced clathrin-mediated endocytosis in living cells [33,35]. It has been demonstrated that GC-SPR is a highly versatile platform for expanding the application of SPR in the IR region [36]. Simply by changing the incident angle, the resonance can be tuned within the range of 1.2 to 2 μm. A GC-SPR cell assay investigating the proliferation kinetics of Hep-2 laryngeal carcinoma cells showed approximately 50% higher detection sensitivity in the 2 μm region than the one obtained in the 1 μm region [37]. Cell coverage in another study [38 yielded comparable results, although they referred to the 0.6–1 μm range.

In the mid-infrared (MIR) spectral region, Surface-Enhanced Infrared Absorption (SEIRA) is an effective method for bio-diagnostic assays. This method enables the determination of the vibrational levels and dynamics of complex molecules during interaction. In [38], the method was used to verify the penetration mechanism of functionalised nanoparticles into cells. In [39], the interaction virus/membrane was studied, with tBLM simulating a cell membrane and the IAV virus as a model.

### 2.8. Dual-Evanescent Mode and Long-Range SPR (Mid-2010s)

In order to extend the detection area of SPR/SPRi sensors beyond the superficial membrane region, SPR configurations exploiting dual-evanescent mode or long-range plasmon propagation were developed [40,41]. Dual-mode SPR differs from long-range SPR (LRSPR) in that it simultaneously excites two plasmonic modes with distinct penetration depths, enabling parallel detection of membrane-proximal and intracellular refractive index changes.

The concept of LRSPR was initially formulated theoretically by [42] and was later developed experimentally [43]. LRSPR is achieved by adding dielectric buffer layers between the metal film and prism to enhance the propagation length of surface plasmons, thereby improving spectral sharpness and detection sensitivity.

In live-cell research, LRSPR enables observation of cellular micromotion and cytoplasmic refractive index fluctuations, as well as early intracellular signalling events [44]. Although fabrication precision is demanded, these systems have shown strong potential for detailed cell physiology studies and high-resolution biomolecular tracking.

The first review article discussing the application of dual-mode SPR in biological research was [6]. Subsequently, this technique was combined with fluorescence analysis and applied for detection of mass redistribution and fluorescence for pathway-specific signalling [45].

LRSPR and dual-evanescent configurations provide higher spectral resolution and signal-to-noise ratio, ideal for monitoring both surface adhesion and intracellular refractive index changes. Though not yet widely applied, LRSPR is increasingly used in mechanistic studies of living cells, offering insights into cytoskeletal remodelling and vesicular transport [44].

### 2.9. Grating-Coupled SPR (GC-SPR)

**Figure 6:**
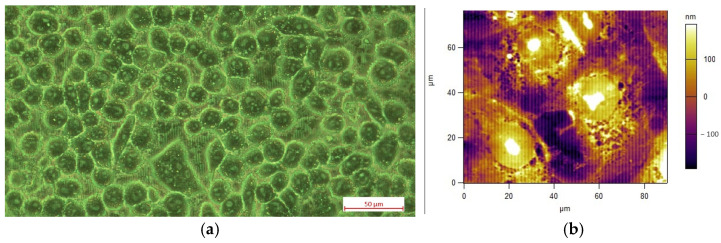
A cell monolayer on the grating: (**a**) optical microscopy image of the monolayer; (**b**) AFM image of the cells on the grating.

GC-SPR uses a periodic metal grating instead of a Kretschmann prism to provide an additional pulse in the plane and excite surface plasmons. The cells are seeded onto the diffraction surface at a specific density. After a growth period, cell monolayers form and are used for experiments. Figure 6 shows a cell monolayer on a diffraction grating along with an atomic force microscope (AFM) image of the cells.

The first report on a cell-based GC-SPR assay was [46]. The resonance shift was measured using phase detection [31]. This technique enables measurements to be taken with a high signal-to-noise ratio. This solves the main problem of GC-SPR detection—excitation of resonance by illumination through the analyte. It also enables controlled flow of the analyte via a microfluidic system.

A different approach was demonstrated in [47]—sequential measurements of the SPR signal were taken at fixed time points after cell seeding, corresponding to the various stages of the cell life cycle. However, this requires prior investigation of the SPR signal induced by the morphological changes and cell proliferation. These factors taken into account, viral infection kinetics [48] and toxicity and efficacy of antiviral drugs [49] can be accurately determined.

One practical advantage of GC-SPR is that the plasmon wavelength can be easily tuned from the visible to the near-infrared region by scanning the wavelength/illumination angle on the grating. This allows penetration depth adjustment and sensitivity optimisation for working with cells.

The GC-SPR sensor platform has several other advantages that make it a promising solution for a range of applications:

- Compact geometry suitable for portable and point-of-care biosensors.
- Ease of multiplexing and on-chip fabrication via nanoimprint lithography or interference lithography.
- Compatibility with transparent substrates, allowing combination with fluorescence microscopy.
- Reduced cost and potential for mass production.

Recent developments in polymer-based gratings and flexible plasmonic films have improved uniformity and biocompatibility, enabling direct live-cell imaging on grating substrates. Additionally, hybrid designs integrating gratings with microfluidics or nanohole structures have achieved sensitivities approaching those of the Kretschmann-type systems while retaining compactness.

### 2.10. Multiparametric SPR (2010s)

Despite the proven effectiveness of label-free techniques such as SPR in biomolecular research, difficulties are encountered in the application of such methods to cellular analysis [50]. The inability to establish a correlation between the signal and the response of a specific receptor subtype represents a significant challenge in this context. This limitation can be overcome by conducting a multiparametric SPR (MP-SPR) analysis. The latter is capable of tracking not only the time dependence of the resonance position but also the minimum peak intensity, the resonance width, and the steepest decline in intensity. This has enabled the precise dissection of the G-protein-coupled receptor pathway [5], as well as the elucidation of details regarding the structure and growth phases of bacterial biofilms [6].

MP-SPR is a particularly powerful method of cellular analysis where morphological changes accompany biochemical signalling, as it provides kinetic and structural information. It is used to monitor endothelial cell adhesion, receptor-mediated signalling and the kinetics of viral entry into living systems. By capturing information on both intensity and phase simultaneously, MP-SPR can distinguish mass loading from morphological responses—a critical distinction for interpreting changes in the cellular refractive index [1]. Careful control of experimental conditions and the use of more sophisticated signal analysis methods are essential in MP-SPR for unambiguously identifying the signalling pathways under investigation.

### 2.11. Coupling with Complementary Techniques (2010s–2020s)

Integrating SPR with other analytical methods, as well as MP-SPR, aims to enhance the interpretive capabilities of cell-based assays. Combined systems, such as SPR–fluorescence [2], SPR–mass spectrometry (MS) and SPR–impedance spectroscopy [51], enable the simultaneous monitoring of physical, chemical and electrical reactions within the same biological process.

**Figure 7:**
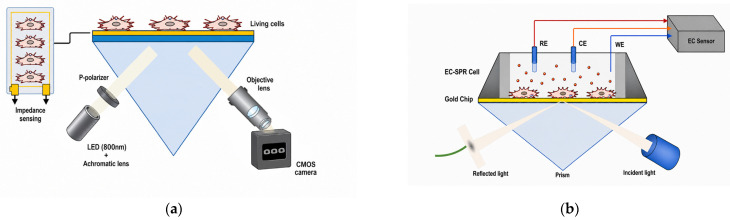
SPR cell assay integrated with: (**a**) impedance sensor—a glass slide coated with comb-shaped thin gold interdigitated electrode. Adapted from Ref. [52]; (**b**) electrochemical sensor. Adapted with permission from Ref. [53]. Copyright 2015 American Chemical Society.

Figure 7a illustrates the principle of integrating an impedance sensor and SPRi into a unified device. SPRi provides information on the distribution of lipids and proteins near the membrane of a living cell within the region of plasmon wave penetration. Concurrently, the impedance sensor provides information on cell coverage and morphology. The authors stated that multiparametric analysis using each of the sensors is necessary for the reliable signalling pathway identification.

To study the effect of daunorubicin treatment on live cancer cells in real time, an electrochemical sensor was coupled to a conventional SPR system, as shown in Figure 7b [53]. The analysis of the data from the two sensors enabled identification of the metabolic processes that determine the SPR signal. It was found that the SPR signal was linearly correlated with the percentage of cell survival—thus, the electrochemical study provided assessment of the potential therapeutic efficacy of bioactive agents. A thorough evaluation that extensively explores the potential of multiple analytical methods coupled to SPR can be found in [52].

Although complementary techniques share the same objectives as MP-SPR, their methodologies are fundamentally different. While MP-SPR provides methods to unlock the potential of SPR detection, complementary techniques employ alternative approaches. Due to the more complex experimental implementation of complementary techniques and the signal analysis challenges facing MP-SPR, only future developments will reveal which approach is superior.

## 3. Summary of Chronological Evolution

The conventional SPR setup has evolved into modern nanoplasmonic, multiparametric, and in situ configurations. SPR has become a universal platform for real-time, label-free cell analysis. Each technological advancement implemented in the techniques listed above has expanded the range of measurable biological phenomena, enabling the study of the physiology of whole cells. Conventional and imaging SPR are the preferred methods for cell analysis. Other techniques, such as MP-SPR, LRSPR, and nanoplasmonic systems, aim to enhance sensitivity and selectivity. They are all based on the detection of changes in the refractive index (RI) in the evanescent field on the gold surface. The SPR sensor detects molecules in and around the plasma membrane of cells on a sensor chip. Consequently, the SPR signal is influenced by the degree of cell coverage and the morphological changes within the cell. Various techniques aim to differentiate these signals. It has been demonstrated that RI changes drastically in response to exogenous stimuli. Studying the detailed mechanisms of processes in living cells depends on the accuracy and sensitivity with which changes in RI are detected. This change is generally estimated to be around 0.0004, and the sensitivity of a conventional SPR sensor is sufficient to detect responses in living cells. However, this is insufficient for some studies, such as those involving pharmacokinetics. Binding of small molecules is detected via the signal generated by larger ligands rather than small antagonists. This requires increased sensitivity, so MP-SPR, LRSPR, phase-sensitive SPR and GC-SPR are considered promising solutions. However, it should be noted that the sensitivities reported in the literature for purified molecules are unlikely to correspond to those observed in cell-based assays. Similarly, the frequently cited RI detection limit of around 10 pg/mm^2^ should be treated with caution.

The distribution of the refractive index within the cell attached to the chip surface determines the varying efficiency of SPR and forms the image during SPRi detection. The image contrast in SPRi is equivalent to the RI detection sensitivity in conventional SPR. In SPRi, sensitivity is determined by the signal-to-noise ratio and the dynamic range of the CCD array, as well as the image processing software. The spatial resolution depends on the optical configuration and can reach several micrometres.

Although the issue of sensitivity in RI measurements can be resolved, the main problem of selectivity still remains, i.e., the ability to distinguish between the sources generating the SPR signal. Possible solutions are discussed in the Section 5.

Although numerous advanced SPR configurations have been demonstrated, only a few platforms are commercially available for routine cell-based assays. These include SPR imaging systems (Horiba OpenPlex—HORIBA (Austria) GmbH—Tulln. Kaplanstrasse 5, A-3430 Tulln, Austria/XelPleX-HORIBA FRANCE SAS, Lille Office. 455, Avenue Eugène Avinée, 59120 LOOS, France/IBIS MX96—IBIS Technologies B.V. Pantheon 5, 7521 PR Enschede, The Netherlands) and multiparametric SPR instruments (Bionavis MP-SPR Navi—BioNavis Ltd., Hermiankatu 6-8 H, 33720 Tampere, Finland), which provide temperature control, microfluidics, and surface chemistries suitable for live-cell measurements. More specialised approaches such as LRSPR, dual-mode SPR, and nanoplasmonic architectures remain primarily laboratory-built and are not offered yet as commercial systems.

## 4. Differentiation by Object of Research

The first section outlines the broad range of SPR techniques employed in cell-based assays. SPR-based assays are adaptable to a variety of technological requirements, making them suitable for a range of research objectives. These objectives include drug screening, viral infection studies and cancer cell characterisation. This section marks a shift from the technological and methodological aspects of SPR toward its applied and diagnostic dimensions. SPR facilitates real-time measurement of the refractive index alterations on the sensor surface, eliminating the requirement for labelling. This allows for applications in various fields of research, including analysis of drug–cell interactions, study of viral infections, and cancer cell characterisation. It is important to note that these applications impose different biological and technical requirements on the SPRi system. For instance, the detection of drug interactions necessitates high-resolution measurements and precise kinetics, while the analysis of viral infection requires biosafe functionalisation of the sensor surface and high sensitivity to viral binding. When studying cancer cell characterisation, it is imperative to distinguish between the different cancer cell phenotypes.

SPR is capable of detecting a wide range of cellular responses, including ligand binding and metabolic processes. However, cell preparation is a prerequisite, involving precise cultivation on the sensor surface and accurate optical tuning.

This section categorises SPR cell-based applications into three principal domains:

- Drug screening;
- Virus–host interaction analysis;
- Cancer cell differentiation and chemotherapeutic response.

It is important to note that an analysis of the SPR cell assay application in the aforementioned areas cannot possibly cover all reported results. Therefore, we have thoroughly reviewed the earliest results reported on the topic, as well as the most recent ones, and have also noted the relevant reviews.

### 4.1. SPR for Drug Screening

The SPR cell-based assay is able to detect real-time physiological changes in cells, enabling the measurement of early and late drug-induced changes. It should be noted that there are only a limited number of articles available that report on the use of SPR-based drug screening methods involving SPR cell-based assays. It is evident that this issue has been ongoing since at least 2008, as evidenced by the review [54] on cell-based, label-free technology in drug discovery, although it makes no mention whatsoever of SPR.

The SPRi cell-based assay has been used to analyse quantitatively the binding kinetics of drug–target interactions and evaluate the molecular origin of drug resistance [52]. Human liver cancer cell line, Hep G2, was cultured on the gold surface and Herceptin-Her2 binding kinetics was measured.

Multiparametric SPR analysis (SPR integrated with an impedance sensor) has been applied in studying the cellular response of MDCK II to the stimulation of G-protein-coupled receptors [55]. The study focused on cell attachment and spreading, as well as cytoskeleton disassembly.

SPRi has been used to assess the potential change in the inner mitochondrial membrane of cancer cells [56]. It is vital to ensure that the membrane potential remains consistent for the anticancer drugs to be effective. Cancer cells were cultivated on an SPR chip with a specific anticancer drug. The cell reaction was monitored in the absence of drugs. A change in the potential related to drug efficacy was observed.

SPRi has been utilised to study the binding kinetics of the monoclonal antibody Herceptin to the human epidermal growth factor receptor 2 (HER2). This kinetics has not been investigated previously. Herceptin was found to be resistant to the sterically induced membrane protein Mucin The-4 [57,58].

In contrast to all other comparable studies in this field, [47,49] utilised a GC-SPR cell-based assay. A novel methodology for evaluating the toxicity and efficacy of drugs was developed. This methodology takes into account the excitation of the plasmon wave when light passes through the analyte (the cells seeded on the diffraction grating). It is imperative to distinguish between the signal induced by morphological changes/cell proliferation and the signal induced by drug action. The study investigated the inhibitory effect and toxicity of chalcones on uninfected and HCoV-229E-infected Vero cells, respectively. A strong correlation was identified in the control studies conducted using MTT assay. The experimental results demonstrated the advantage of GC-SPR, showing higher sensitivity with deeper plasmon wave penetration, easily achieved simply by changing the angle of incidence.

### 4.2. SPR for Virus–Host Interaction Analysis

The SPR technique is widely used for virus detection because, unlike the enzyme-linked immunosorbent assay (ELISA), it does not require labelling, thereby eliminating the need to functionalise multiple antibodies. In addition, it measures binding–unbinding kinetics with high sensitivity, ensuring reliable virus detection. There are numerous articles dedicated to the detection of various viruses. Some of the studies on diagnosing SARS-CoV-2 can be found in [59,60].

With regard to the SPR cell-based assay, its first application for studying the kinetics of virus-infected cells was reported in [48]. The aim of the study was to demonstrate its applicability to the kinetic analysis of early stages of viral infection of cells and to verify the activity of antiviral drugs in infected cells. The SPR signal demonstrated the growth kinetics of Vero 6 cells infected with HCoV-229E. It was found that there was complete agreement with the results of the control experiment conducted using the MTT assay. A significant limitation of the cellular SPR assay in studying virus–host kinetics was noted—namely, that it cannot be applied for a period exceeding two virus replication cycles. This limitation is due to the cytopathic effects induced by the virus, which disrupt cell adhesion and generate debris that destabilises the SPR signal. This makes the long-term monitoring after the early stages of infection unreliable.

### 4.3. SPR for Cancer Cell Characterisation

The effectiveness of the SPR cell assay is determined by two specific advantages: real-time detection and label-free detection. The first allows for: (i) monitoring of cancer cell responses to chemotherapeutic stress; (ii) quantification of drug–target interactions in intact cancer cells to understand resistance mechanisms.

Label-free detection enables the detection of changes in the refractive index (RI), allowing for detection of early apoptosis, necrosis, and morphological remodelling—key indicators of drug sensitivity.

#### 4.3.1. Cell Adhesion and Morphological Profiling

Following the demonstration that SPR detection is not limited to changes in cell adhesion and ligand binding to the cell membrane (see reference [1]), Yanase’s group applied SPRi to investigate the response of RBL-2H3 cancer cells, PAM212, and A431 to treatment with EGF (see reference [61]). This approach was later extended to cSPR-based detection of apoptosis-related changes, including morphological changes, cell detachment, and refractive index shifts during apoptosis [62]. It was found that subtle morphological changes are often detected before they become visible through conventional microscopy or metabolic analyses.

#### 4.3.2. Chemotherapeutic Response

The results reported in [16] demonstrated the potential of the SPR cell-based assay for real-time detection and/or diagnosis of malignant tumours [63]. This finding was supported by the well-established correlation between antigen-induced intracellular signalling events and alterations in the SPR signal. RBL-2H3 cells were cultivated on the SPR chip and exposed to a series of molecules, including Syk, SLAP, and LAT. Subsequent analysis using SPR [9] confirmed its ability to detect cancer cells. A new experimental strategy based on the SPR cell assay was employed to identify a novel anticancer target in the MCF-7 breast cancer cell line [64].

## 5. Discussion

The advantages and disadvantages of SPR, as observed in conventional studies of biomolecular binding kinetics, manifest themselves in various ways when applied to cellular analyses. Selectivity issues are primarily attributable to non-specific reactions caused by the embedded matrix. As proposed in [65], in conventional application, the ligand immobilisation can be performed without the use of intermediary molecules. Then, selectivity can be determined solely by the selectivity of the ligand. Such a solution is possible in biochemical reactions because the resonance shift occurs as a result of ligand/analyte binding, which causes accumulation of molecular mass in the evanescent field region. In cell analysis, the SPR signal is due to redistribution of intracellular mass, or changes in cell layer confluence, or changes induced by external stimuli. This raises the question of whether, and to what extent, SPR technology can enable selective detection in cell-based assays.

To some extent, SPR cell assays were adapted for the selective detection of specific cellular responses by multiparametric analysis [4,5,6]. This approach involves tracking not only the time dependence of the resonance position, but also that of the peak minimum intensity, resonance width and steepest fall intensity. The drawback is the necessity of a preliminary study to identify the pair signalling pathway/SPR signal. There are two alternatives to multiparametric analysis. The first is SPR coupling with complementary techniques (see Section 2.11). The second involves an experimental protocol in which appropriate control groups are defined. The second approach is effective for studying the kinetics of cellular processes. As pointed out in [66], an increase in the cell coverage of the sensor surface leads to an increase in the resonance shift. The changes in the cell coverage on the SPR slide can be attributed to two main factors: an increase in cell number due to cell growth or to morphological changes in the cell layer. In order to eliminate the first factor, Ref. [67] analysed the SPR signal after the formation of a confluent monolayer of cells and following drug treatment. This does not mean that time-resolved signals provide information about specific changes to the cell caused by external factors. Another detection method is required to provide an additional channel of information (see Section 2.9). We will discuss the possibility of obtaining all this information using SPR technology.

In an SPR-based assay, selectivity can be defined as the ability to recognise the activity of specific cellular structures. Until now, the SPR signal has been determined by the response of the entire cell, which is obtained through contact between the sensor surface and the cell membrane. It may be worthwhile considering the possibility of studying subcellular organelles solely through SPR detection. While this approach cannot match the spatial resolution of SPR microscopy, it would provide valuable insights into pharmacokinetics.

In this regard, it is important to explore the possibilities for increasing the sensing range of the SPR technique. The applications of IR SPR discussed in [33,35] do not fully reflect the potential of the method. Detection was carried out in the near-IR region, where the plasmon wave penetration depth is around 700 nm. In the 2–2.5 μm range, the penetration depth is 1–2 μm, enabling the study of events in the cell cytoplasm, but not the nucleus. In the 4–6 μm range, the penetration depth is 5–10 μm; this is comparable to the size of a cell and allows organelles to be detected [34]. Despite the promising prospects, no significant research has yet been conducted in this field. In the context of the Kretschmann configuration, the reason for this was presumed in [34]: the lack of laser sources and optical components to extend the application of SPR into the IR range. Despite the research conducted in the subsequent years [68,69,70,71], no significant progress was reported.

GC-SPR applications open up new opportunities for infrared research. A key advantage is that the resonance wavelength can be easily controlled by simply adjusting the angle of incidence. As mentioned in Section 2.7, plasmons can be excited within the 2-micrometre range. Maintaining deep resonance over a wide spectral range requires optimisation of the structure, including the periodicity, profile and depth of the grating, the thickness of the plasmonic coatings and the type of substrate material. It should be noted that this is no easy task, particularly given the challenges involved in gratings manufacturing. The GC-SPR cell analysis described in [37] was based on gratings with a rectangular profile. This enables SPR measurements to be performed in two modes. One plasmon is sensitive to cell proliferation, while the other is used as a reference. New approaches could be focused on applying dual-mode operation in the IR range. Firstly, one might expect an increase in accuracy, which would eliminate the need for control groups. Secondly, controlling the penetration depth by adjusting the angle of incidence could be combined with time-resolved signal measurement. This could provide information on the temporal and spatial localisation of processes around the cell membrane, taking penetration depth into account.

The most suitable spectral window for IR-SPR cell assays is 4–6 μm. Achieving operation in this range is associated with challenges related to manufacturing gratings with a period of approximately 5 μm. However, they can be overcome, as demonstrated in [71]. This would lead to technical breakthroughs in terms of spatial detection range and sensitivity. Scanning cells through controlled penetration of plasmonic waves is expected to enable real-time monitoring of the proliferation observed in the cell volume covered by the plasmonic field. This will significantly advance the development of new drugs, in particular.

## 6. Conclusions

Over the past 20–25 years, significant development has occurred in the use of SPR techniques for cell-based assays. The driving force behind this evolution has been the need for accurate, comprehensive and reliable investigation of biochemical reactions in their natural physiological environment.

An analysis of this development revealed the need to apply SPR technology in field-based analysis. The driving forces behind this are utilising the full potential of SPR detection (multiparametric analysis), expanding these capabilities (SPRi) and identifying specific signalling pathways by integrating other measurement methods.

The analysis outlined the role and potential of the GC-SPR cell assay. The ability to tune the resonance in the 2 µm range enables control of the penetration depth. When combined with time-resolved signal measurement, this could provide information on the temporal and spatial localisation of processes around the cell membrane. This would enable subcellular organelles to be studied solely through SPR detection, providing valuable information on virus–host interaction and pharmacokinetics.

## Data Availability

Dataset available on request from the authors.

## Abbreviations

The following abbreviations are used in this manuscript:

SPR	Surface plasmon resonance
cSPR	Classical SPR
SPRi	SPR imaging
CCD	Charge-coupled device
CMOS	Complementary metal-oxide-semiconductor
EOT	Exceptional optical transmission
IR	Infrared
Mid-IR	Mid-infrared
FTIR	Fourier Transform Infrared Spectroscopy
SEIRA	Surface-Enhanced Infrared Absorption
LRSPR	Long-range SPR
GC-SPR	Grating-coupled SPR
AFM	Atomic force microscopy
MP-SPR	Multiparametric SPR
SPR-MS	SPR–mass spectrometry
ELISA	Enzyme-linked immunosorbent assay
MTT	3-(4,5-dimethylthiazol-2-yl)-2,5-diphenyltetrazolium bromide assay
RI	Refractive index
RIU	Refractive index unit
LAT	Linker for activation of T cells
